# Integrated Piezoelectric Vibration and In Situ Force Sensing for Low-Trauma Tissue Penetration

**DOI:** 10.34133/cbsystems.0417

**Published:** 2025-10-21

**Authors:** Bingze He, Yao Guo, Guangzhong Yang

**Affiliations:** ^1^ Institute of Medical Robotics, School of Biomedical Engineering, Shanghai Jiao Tong University, Shanghai, China.; ^2^ Shanghai Key Laboratory of Flexible Medical Robotics, Tongren Hospital, Institute of Medical Robotics, Shanghai Jiao Tong University, Shanghai, China.

## Abstract

Precision-controlled microscale manipulation tasks—including neural probe implantation, ophthalmic surgery, and cell membrane puncture—often involve minimally invasive membrane penetration techniques with real-time force feedback to minimize tissue trauma. This imposes rigorous design requirements on the corresponding miniaturized instruments with robotic assistance. This paper proposes an integrated piezoelectric module (IPEM) that combines high-frequency vibration-assisted penetration with real-time in situ force sensing. The IPEM features a compact piezoelectric actuator integrated with a central tungsten probe, generating axial micro-vibration (4,652 Hz) to enable smooth tissue penetration while simultaneously measuring contact and penetration forces via the piezoelectric effect. Extensive experiments were conducted to validate the effectiveness and efficacy of the proposed IPEM. Both static and dynamic force-sensing tests demonstrate the linearity, sensitivity (9.3 mV/mN), and accuracy (mean absolute error < 0.3 mN, mean absolute percentage error < 1%) of the embedded sensing unit. In gelatin phantom tests, the module reduced puncture and insertion forces upon activation of vibration. In vivo experiments in mouse brains further confirmed that the system could reduce penetration resistance (from an average of 11.67 mN without vibration to 7.8 mN with vibration, decreased by 33%) through the pia mater and accurately mimic the electrode implantation–detachment sequence, leaving a flexible electrode embedded with minimal trauma. This work establishes a new paradigm for smart surgical instruments by integrating a compact actuator–sensor design with real-time in situ force feedback capabilities, with immediate applications in brain–machine interfaces and microsurgical robotics.

## Introduction

**Fig. 1.**
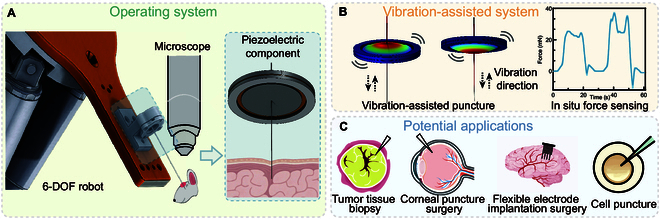
Schematic illustration of the proposed vibration-assisted puncture and in situ force-sensing system. (A) A 6-DOF robotic arm integrated with a microscope and a piezoelectric puncture module enables precise manipulation and real-time observation of soft tissue penetration. (B) The piezoelectric component simultaneously provides axial vibration for membrane puncture and real-time in situ force sensing based on piezoelectric coupling. (C) The system has potential applications in various biomedical procedures, including tumor tissue biopsy, corneal puncture surgery, flexible neural electrode implantation, and single-cell puncture.

Precision penetration of biological membranes with minimal damage during functional device implantation is essential for diverse biomedical applications, from in vitro procedures such as intracytoplasmic sperm injection (ICSI) and microinjection into single cells [1], to in vivo operations including drug delivery, neural electrode implantation, and retinal microsurgery [2–4]. These applications are illustrated in Fig. 1C and the demand for reliable and minimal damage bio-membrane penetration technologies continues to grow. For instance, in deep brain stimulation and brain–computer interface (BCI) research, neuro-electrodes must be inserted across multiple layers of brain tissue (dura mater, pia mater, and cortical matter) without rupture or causing excessive inflammation [5,6]. In ophthalmic surgery, precise puncture of delicate layers such as the cornea and retina is essential to avoid vision impairment [7]. Likewise, in minimally invasive cancer diagnostics and soft tissue biopsies, low-force membrane penetration plays a vital role in preserving surrounding structures [8]. These scenarios underscore a shared technical challenge: the need to puncture thin, tough, or compliant membranes at micro-scale while avoiding collateral tissue damage, excessive mechanical force, or misalignment during insertion. To address this challenge, conventional approaches mainly employ sharp-tipped microprobes or micro-tools actuated through manual or rapid motorized control. These techniques frequently necessitate high-velocity insertions to surpass tissue rupture thresholds, potentially leading to increased inflammation and hemorrhaging—particularly when operating on delicate neural tissues [9]. Furthermore, due to biological tissue heterogeneity and the complex mechanics of rupture, the insertion force profile is not readily predictable, which limits the development of reliable real-time sensing systems and adaptive control strategies and ultimately compromises procedural precision and safety.

To reduce penetration force while maintaining precision, research interests have recently focused on the development of vibration-assisted and ultrasonic-assisted insertion techniques, empowered with bioinspired principles. High-frequency axial vibrations, either through piezoelectric actuation or ultrasonic transducers, have been shown to significantly lower puncture and insertion resistance by promoting dynamic tissue rupture and reducing sliding friction [10–12]. For instance, Johnson et al. [13] proposed a piezo-drill probe capable of cell membrane penetration through axial vibration without relying on large axial forces. Similarly, ultrasonically actuated neural probes demonstrated improved insertion into mouse brains with less trauma and reduced glial scarring [14]. Bionic designs mimicking mosquito proboscis motion also benefit from micro-vibrations to facilitate low-resistance, high-precision penetration [15]. Song et al. [16] proposed a novel biopsy capsule robot that employs a rotating high-speed blade strategy for soft tissue scraping, aiming to avoid the tissue tearing issues inherent in existing biopsy methods. However, these vibration-based tools lack integrated force-sensing capabilities, limiting their ability to adaptively modulate insertion behavior or detect membrane rupture events in real time. Moreover, most systems are restricted to open-loop control with no feedback from the tip–tissue interface, which compromises safety and precision. Integrating force sensing would enable the detection of subtle mechanical cues, such as the moment of membrane rupture or changes in insertion resistance, thereby facilitating dynamic adjustments of insertion trajectory, speed, and tool configuration.

Previous studies have demonstrated the potential of force-based feedback in neural and ophthalmic surgeries [17,18]. For example, during deep brain electrode implantation, a sudden drop in insertion force typically signals dura or pia penetration, which can be used to prevent over-insertion or misplacement [15]. Similarly, retinal surgical instruments equipped with force sensors maintain the tip force and scleral force within a predetermined narrow range to avoid retinal damage [19,20]. However, implementing force sensing in microscale biomedical procedures is inherently challenging. Conventional commercial sensors are often too bulky or lack sufficient sensitivity to detect sub-millinewton- or micronewton-level forces. In addition, external sensing configurations also suffer from compliance losses, cable disturbances, or spatial offset from the actual tool tip. Recent progresses in embedded or tip-integrated sensors offer promising solutions, including piezoresistive [21], optical fiber [22], and capacitive sensing [23] strategies. Nonetheless, these methods usually require additional readout electronics, sacrifice mechanical compactness, or are limited in dynamic response speed—especially under rapid puncture or vibration-assisted insertion. Notably, the integrated actuation and sensing in soft robots has been proven to enable intelligent control through feedback while maintaining the compactness of the hardware system [24].

Thus far, new developments in piezoelectric self-sensing actuation offer a potential solution to bridge this technical gap. Because of the duality of the piezoelectric effect—where mechanical deformation generates electric charge (direct piezoelectric effect) and applied voltage induces mechanical motion (inverse piezoelectric effect)—the same element can be used both as an actuator and as a sensor [25–27]. In nanopositioning systems, piezoelectric self-sensing has been extensively explored for displacement and force estimation [28]. These methods exploit charge measurement from the piezoelectric element to estimate external loading conditions, eliminating the need for additional force sensors and minimizing system complexity. However, most existing works focus on static or low-dynamic applications, such as Micro-Electro-Mechanical Systems (MEMS) or nanopositioners, rather than high-speed, biologically relevant puncture events.

In this study, we propose a novel integrated piezoelectric module (IPEM) based on a piezoelectric ceramic element designed specifically for bio-membrane puncture and insertion applications (see Fig. 1A). By leveraging the inverse piezoelectric effect, we induce high-frequency axial vibrations at the probe tip to assist membrane rupture and reduce insertion resistance. Simultaneously, using the direct piezoelectric effect, we measure the generated charge response during tissue interaction, enabling real-time force sensing without additional hardware (Fig. 1B). As illustrated in Fig. 1C, the system has potential applications in various biomedical procedures, including tumor tissue biopsy, corneal puncture surgery, flexible neural electrode implantation, and single-cell puncture. This dual-functionality design addresses several key challenges in existing systems:

1. Reduced penetration force: Axial micro-vibrations enhance rupture efficiency and reduce peak insertion forces, while maintaining low transverse displacement to preserve accuracy and minimize off-axis trauma.
2. Accurate in situ force monitoring: The same piezoelectric element serves as a charge-based force sensor, capturing puncture events and insertion resistance profiles in real time, even during high-frequency operation.
3. Compact and minimalist architecture: By avoiding external sensors, bulky load cells, or separate vibration modules, the device offers an integrated, lightweight platform suitable for small-scale biomedical procedures.

## Materials and Methods

### Probe–tissue interaction models

This section presents the mechanical models for the interaction between a rigid probe and soft tissue during insertion, under nonvibrating and high-frequency axial vibrating conditions. The insertion process is divided into pre-puncture (contact phase), puncture, and post-puncture (insertion phase).

#### Nonvibrating insertion model

In a nonvibrating condition, the probe inserts into the tissue at a constant velocity $v_{\mathrm{ins}}$.

**Fig. 2.**
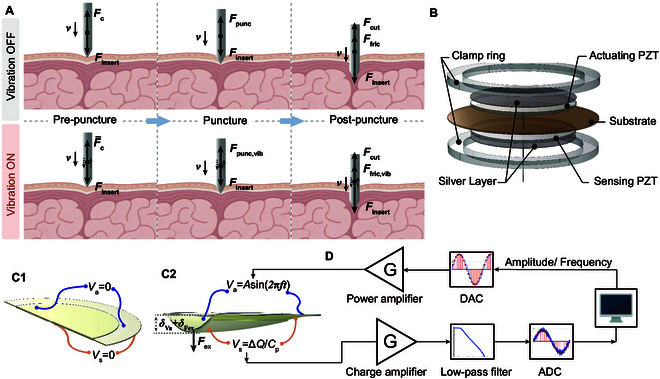
Working principle and structural design of the integrated piezoelectric puncture and sensing system. (A) Schematic comparison of the puncture process with and without axial vibration, including 3 stages: pre-puncture (contact compression), puncture (membrane rupture), and post-puncture (insertion into tissue). (B) Exploded view of the piezoelectric actuator–sensor integration, consisting of a substrate, an actuating PZT, and a sensing PZT, clamped together by a ring and coated with silver electrodes. (C1 and C2) Working mechanism of the piezoelectric sensor: (C1) under no external load, no voltage is generated; (C2) under external force while the actuator applies a sinusoidal voltage. (D) Signal flow and circuit design for vibration driving and force sensing. The upper loop represents the actuation path using a DAC and power amplifier, while the lower loop shows the sensing path using a charge amplifier, low-pass filter, and ADC for real-time acquisition of force signals.

Before puncture, the probe tip indents the tissue, generating an elastic contact force $F_{c}$(as shown in Fig. 2A. At this point, the probe is subjected to a vertical downward insertion force $F_{\text{insert}}$ and a vertical upward $F_{c}$ in the vertical direction, and the values of the two are equal.

$$F_{\text{insert}}=F_{c}$$ (1)

This interaction is modeled as a conical indenter pressing into an elastic half-space, based on hertz-like contact mechanics [29]. The contact force $F_{c}$ is given by:

$$F_{c}=\frac{2E_{t}\text{tan}\;\theta_{\mathrm{hc}}}{\pi \left(1-\nu_{t}^{2}\right)}\delta^{2}$$ (2)

where $E_{t}$ is the tissue’s Young’s modulus, $\nu_{t}$ is the Poisson’s ratio, $\theta_{\mathrm{hc}}$ is the probe tip’s half-cone angle, and δ is the indentation depth. The quadratic relationship reflects the geometry of the conical tip. Tissue viscoelasticity may slightly increase the effective stiffness at high indentation rates; the process is approximated here as quasi-static to first order.

Puncture occurs when the stress at the probe tip exceeds the tissue’s rupture threshold. Using an energy balance approach [30], the puncture force $F_{\text{punc}}$ is expressed as:

$$F_{\text{punc}}\approx E_{t}\sqrt{\frac{\Gamma }{E_{t}d}}$$ (3)

where Γ is the fracture toughness per unit area, and d is the characteristic crack length at the probe tip. The puncture force typically represents the peak of the contact force curve, marking the maximum force required before the tissue ruptures. After puncture, the contact force drops rapidly.

Post-puncture, the probe advances through the tissue, with resistance primarily from friction between the probe shaft and the tissue. The insertion force is:

$$F_{\text{insert}}=F_{\mathrm{cut}}+F_{\text{fric}}$$ (4)

where $F_{\mathrm{cut}}$ is the residual cutting force at the tip (typically small after puncture) and $F_{\text{fric}}$ is the frictional force exerted by the tissue section on the probe, expressed as:

$$F_{\text{fric}}=\text{μp}\text{π}\text{dL}$$ (5)

where μ is the friction coefficient, p is the normal pre-stress from the tissue’s constitutive properties, d is the probe shaft diameter, and L is the insertion depth. Viscoelasticity reduces the effective friction coefficient $\mu_{\mathrm{eff}}$ at higher insertion velocities, as the tissue has less time to conform to the probe shaft [31].

#### Vibrating insertion model

In vibrating conditions, the probe maintains a constant insertion velocity $v_{\mathrm{ins}}$ while superimposing high-frequency axial vibrations (frequency f, amplitude A).

The instantaneous indentation depth varies as $\delta \left(t\right)=\delta_{0}+$$A\text{sin}\left(2\pi \mathrm{ft}\right)$. Due to the rate-independent nature of elastic contact, the average contact force ${\bar{F}}_{c}$ is similar to the nonvibrating case [29]:

$${\bar{F}}_{c}=\frac{2E_{t}\text{tan}\;\theta_{\mathrm{hc}}}{\pi \left(1-\nu_{t}^{2}\right)}\delta_{0}^{2}$$ (6)

where $\delta_{0}$ is the mean indentation depth. Given the small amplitude ($A\ll \delta_{0}$), the effect on the ${\bar{F}}_{c}$ is negligible. However, rapid contact/separation cycles may induce localized stress spikes.

Vibration promotes crack initiation through dynamic stress concentration, reducing the effective puncture force $F_{\text{punc},\mathrm{vib}}$ [29,30]. The puncture force is modeled as:

$$F_{\text{punc},\mathrm{vib}}\approx \alpha \left(f,\;A\right)F_{\text{punc}}$$ (7)

where $\alpha \left(f,\;A\right)<1$ is a reduction factor that decreases with increasing frequency f and amplitude A. The oscillatory motion induces transient stress peaks, facilitating micro-crack propagation at lower average loads. Post-puncture, the probe continues to advance through the tissue under high-frequency vibration. The insertion force $F_{\text{insert},\mathrm{vib}}$ is

$$F_{\text{insert},\mathrm{vib}}=F_{\mathrm{cut}}+\mu_{\mathrm{vib}}\text{p}\text{π}\text{dL}$$ (8)

where $\mu_{\mathrm{vib}}$ is the effective friction coefficient under vibration, and $\mu_{\mathrm{vib}}<\mu$. According to vibration-assisted friction theories (e.g., LuGre model), high-frequency motion dynamically lubricates the interface, leading to $\mu_{\mathrm{vib}}<\mu$ [32]. Additionally, during vibration, the tissue has insufficient contact time to fully conform to the probe shaft, reducing the average shear force [33]. Enhanced dynamic tissue failure further lowers the cutting resistance at the tip.

### Fundamental principles of piezoelectric effect

The piezoelectric effect describes the bidirectional coupling behavior of certain crystalline materials, which generate electric charge when subjected to mechanical stress (direct piezoelectric effect), and conversely produce mechanical strain upon application of an electric field (inverse piezoelectric effect). This coupling can be expressed by the following linear constitutive equations:

$$\left\{\begin{array}{cc} \;S=s^{E}T+d^{t}E & \\ \;D=dT+\varepsilon^{T}E & \end{array}\right.$$ (9)

where S and T denote strain and stress tensors, E and D represent electric field and electric displacement vectors, $s^{E}$ is the elastic compliance matrix under constant electric field, $\varepsilon^{T}$ is the dielectric permittivity matrix under constant stress, and d is the piezoelectric coefficient matrix. In a simplified one-dimensional form, these relations reduce to the inverse piezoelectric effect S=dE and the direct piezoelectric effect D=dT. (Section S1 provides a detailed introduction to the relevant principles.)

For common piezoelectric ceramics such as lead zirconate titanate (PZT), the $d_{33}$ coefficient along the polarization direction typically ranges from 300 to 600 pC/N (Table S1 summarizes the physical parameters of PZT-5H ceramics), enabling micrometer-scale actuation displacement and sensitive detection of small contact forces. This makes piezoelectric materials well suited for low-frequency, micro-force, and bio-compatible in situ sensing applications. In this work, the piezoelectric effect is employed to realize simultaneous high-frequency axial vibration actuation and force sensing during probe insertion into biological tissue. A high-frequency excitation voltage induces axial micro-displacement of the piezoelectric actuator, assisting penetration, while a separate piezoelectric element measures stress-induced charge signals, enabling in situ force sensing without external sensors.

### IPEM structural design

The IPEM designed herein integrates the drive and sensing functions into a compact assembly, as illustrated in Fig. 2B. The device uses 2 PZT discs aligned along a central axis, each poled through its thickness. One PZT disc serves as the actuator. A drive voltage is applied to make it expand or contract. The other PZT disc is the sensor, held under the same mechanical constraints so that any axial force produces strain on it and yields charge via the direct effect. A sharp tungsten probe is rigidly attached along this axis and transmits contact force to the discs. Two metal clamp rings press the assembly together: they provide mechanical preloading and electrical contact (Section S2 provides a detailed description and Table S2 provides the material properties of the components).

Without applying any excitation voltage or external force, the upper and lower 2 PZTs will not undergo any deformation (as shown in Fig. 2C1). The sensor PZT will not generate an induced voltage (as shown in Fig. 2C2). When the excitation voltage $V_{a}$ is applied to the actuator PZT, strain $\delta_{\mathrm{Va}}$ will occur in the actuator PZT at this time. The actuator PZT drives the probe and sensor PZT to move forward. If the center of the mechanism is subjected to an external axial force $F_{\mathrm{Ex}}$ at this time, a new strain $\delta_{\mathrm{Ex}}$ will occur in the 2 PZTs. At this point, the sensor PZT will sense the charge ΔQ generated by the strain $\delta_{\mathrm{Va}}$ and $\delta_{\mathrm{Ex}}$. The mechanical coupling (through rigid probes and fixtures) ensures the force–strain sensor PZT at the tip. This bonded disc layout allows for in situ driving and sensing simultaneously. Since each piezoelectric element independently follows the forward and inverse relationship, the piezoelectric mechanism designed here integrates the driving and sensing functions into a compact component.

The sensor’s wiring and amplifier are high-impedance so that the actuator drive does not leak into the sensing channel. High input impedance on the sensor amplifier prevents loading the piezo, so that each PZT effectively operates independently. In Fig. 2D, the induced charge ΔQ generated by the sensor PZT is first amplified by a charge amplifier (with detailed design described below) and filtered by a low-pass filter (see Section S3.2), effectively minimizing crosstalk. The signal is then sampled by an analog-to-digital converter (ADC, see Section S3.3) and transmitted to a computer for further analysis. Simultaneously, the computer outputs the desired vibration frequency and amplitude for the actuator PZT. After digital-to-analog conversion (DAC, see Section S3.4) and power amplification (see Section S3.5 and Fig. S2), the signal is applied across the actuator PZT.

### Charge amplifier circuit design and theoretical basis

The piezoelectric sensor outputs an extremely small charge signal, which can be modeled electrically as a charge source in parallel with a high-impedance capacitor. A charge amplifier converts this charge into a measurable voltage via an operational amplifier with a feedback capacitor $C_{f}$ and resistor $R_{f}$, forming an integrator with transfer function (see Section S3.1 and Fig. S1):

$$V_{\text{out}}\left(t\right)=\frac{Q\left(t\right)}{C_{f}}$$ (10)

$$H\left(\mathrm{j\omega}\right)=\frac{1}{C_{f}}\cdot \frac{1}{1+\mathrm{j\omega}R_{f}C_{f}}$$ (11)

where *Q*(*t*) is the input charge, $V_{\text{out}}$ is the output voltage, and $H\left(\mathrm{j\omega}\right)$ is the frequency response of the circuit. The cutoff frequency is determined by:

$$f_{c}=\frac{1}{2\pi R_{f}C_{f}}$$ (12)

Considering the typical puncture force of mouse brain tissue (~10 mN) and the resulting piezoelectric charge magnitude in the picocoulomb range, values of $C_{f}=10\;\mathrm{nF}$ and $R_{f}=100\;\mathrm{G\Omega}$ were selected. This configuration provides a charge-to-voltage gain of 0.1 V/pC and a low cutoff frequency (~0.0016 Hz), ensuring faithful tracking of sub-hertz biological force signals while minimizing the low-frequency drift and noise (Section S3.1 provides a detailed description).

Such a design allows high-fidelity force measurement under the background of high-frequency actuation vibration, facilitating subsequent puncture force analysis, mechanical modeling, and feedback control without additional sensing hardware.

## Results and Discussion

### Vibration performance characterization

To validate the high-frequency axial excitation capability for biological puncture applications, a combination of finite element modal analysis and laser Doppler vibrometry was employed to characterize the system’s vibrational performance and response behavior.

**Fig. 3.**
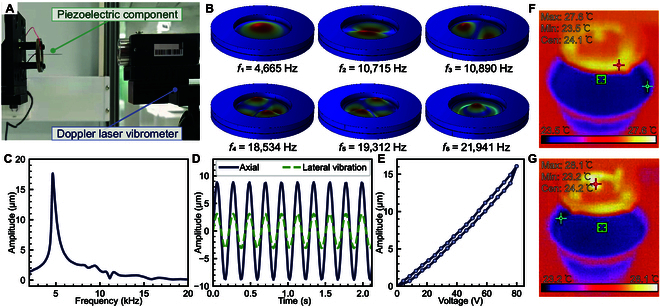
(A) Experimental setup for vibration performance testing. The IPEM is mounted on a precision 3D stage and actuated via a sinusoidal signal. A laser Doppler vibrometer (LDV) is aligned to measure out-of-plane displacement at the probe tip. (B) Finite element analysis (FEA) simulation results showing the first 6 vibration modes of the IPEM structure. The first natural frequency (*f*_1_ = 4,665 Hz) corresponds to dominant axial mode, while higher-order modes exhibit increasing degrees of radial and torsional deformation. (C) Experimental frequency response obtained by sweeping the excitation frequency while measuring vibration amplitude using LDV. A sharp resonance peak appears near 4.6 kHz. (D) Real-time vibration amplitude in axial and lateral directions at resonance. The axial amplitude reaches ±9.6 μm under 80 V sinusoidal excitation, while lateral amplitude remains below ±3.1 μm. (E) Displacement–voltage hysteresis loop of the IPEM at the resonant frequency. (F) Infrared thermograph before vibration, showing IPEM (upper red area, max 27.6 °C) and gelatin phantom (lower blue area, center 24.1 °C). (G) Infrared thermograph after 10 min of vibration with 5 mm probe insertion, showing IPEM–probe junction (max 28.1 °C) and phantom center (24.2 °C), with a temperature rise of 0.5 and 0.1 °C, respectively.

The IPEM was horizontally mounted on a custom-built test rig via 4 peripheral supporting legs, with its axial direction aligned parallel to the table surface. The entire assembly was placed on a vibration-isolated optical table. A Doppler laser vibrometer was positioned in front of the device, and its focal point was carefully aligned with the tip of the tungsten probe to enable precise vibration measurements (Fig. 3A).

Modal analysis was conducted using COMSOL Multiphysics (version 6.1), where the IPEM was modeled as a bonded circular bimorph structure with a central tungsten probe. Material parameters for the PZT ceramics and the tungsten probe were defined based on datasheet values, and mechanical boundary conditions reflected the 4-point fixed constraint. The first 6 natural vibration modes were obtained, with the fundamental mode occurring at approximately 4,665 Hz and exhibiting a symmetric axial drumhead-like deformation (Fig. 3B). Higher-order modes involve complex mixed deformations, including nodal lines and out-of-plane flexural motion, which are less favorable for controlled axial actuation. In the experimental test, a sinusoidal voltage sweep ranging from 2 to 20 kHz was applied to the piezoelectric actuator using a signal generator and a power amplifier. The peak-to-peak voltage of the excitation signal was set at 80 V. Frequency response analysis revealed a distinct resonance peak near 4.6 kHz, consistent with the simulated first-mode frequency, with a maximum axial displacement of approximately 176 μm (Fig. 3C). At the resonant frequency, the axial and lateral displacements were simultaneously recorded using the Doppler laser vibrometer (Fig. 3D). The axial vibration amplitude exceeded ±8.8 μm, while the lateral displacement remained below ±3.1 μm. This dominant axial displacement is critical for minimizing lateral tissue damage during puncture. To assess the voltage–displacement relationship and potential hysteresis, a quasi-static voltage sweep was applied from 0 to 80 V. The measured displacement–voltage curve (Fig. 3E) demonstrated near-linear behavior. A small but noticeable hysteresis loop was observed, with a maximum amplitude deviation of approximately 0.9 μm between the forward and reverse sweeps, corresponding to about 5.6% relative hysteresis, indicating reliable repeatability for controlled actuation.

### Performance evaluation of piezoelectric force-sensing component

**Fig. 4.**
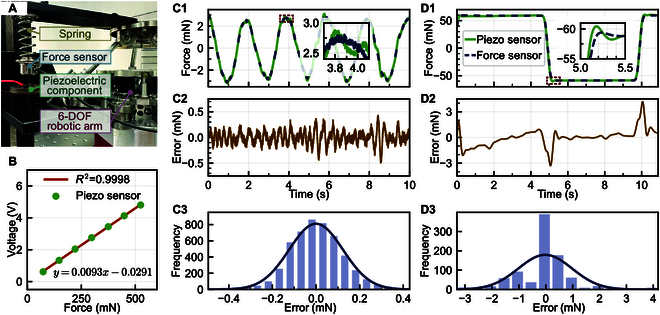
(A) Experimental setup showing a 6-degree-of-freedom (6-DOF) robotic arm applying programmable contact forces via a spring mechanism. The integrated piezoelectric module (IPEM) and a commercial force sensor are mounted in series at the arm’s end-effector to enable synchronous measurement and validation. (B) Static calibration curve of the IPEM, showing excellent linearity between the applied force and output voltage with a coefficient of determination *R*^2^ = 0.9998 and a slope of 9.3 mV/mN. (C1) Force signals recorded by the IPEM and commercial sensor under sinusoidal loading. (C2) Time-domain error under sinusoidal loading, showing peak-to-peak error below ±0.5 mN. (C3) Histogram of sinusoidal tracking errors, approximating a normal distribution centered near 0 with narrow spread. (D1) Force signals recorded by the IPEM and commercial sensor under square-wave loading. (D2) Time-domain error under square-wave loading, with transient error peaks during sharp transitions. (D3) Histogram of square-wave tracking errors, showing larger spread due to dynamic overshoot.

To validate the performance of the developed piezoelectric force-sensing unit under small-force measurement scenarios, a testing platform was designed based on a 6-degree-of-freedom (6-DOF) robotic arm and a spring-loading mechanism. In this platform, the piezoelectric element was connected in series with a commercial force sensor, which served as a high-precision reference standard (Fig. 4A). A calibrated spring mechanism was employed to apply known forces and achieve periodic loading. The platform enabled dynamic force loading with different waveforms (e.g., sinusoidal and square waves), while simultaneously recording force outputs from both the IPEM and the commercial sensor.

To establish the force–voltage conversion relationship of the piezoelectric element, static calibration was conducted (Section S4.1 and Fig. S3 provide a detailed description) under controlled laboratory conditions (temperature: (25 ± 2) °C; relative humidity: 50% ± 10%). By gradually increasing the applied force and recording the corresponding output voltage, a fitted linear curve was obtained (Fig. 4B), showing a high correlation coefficient of *R*^2^ = 0.9998, indicating excellent linearity within the working range. The fitted equation was y=0.0093x−0.0291, where *y* represents the output voltage (in volts), and *x* is the applied force (in millinewtons), resulting in a sensitivity of approximately 9.3 mV/mN.

Dynamic tests were conducted using both sinusoidal and square-wave loading signals. Under sinusoidal loading, the piezoelectric element accurately tracked the force curve of the commercial force sensor (Fig. 4C1), with minimal fluctuation in error over the entire cycle (Fig. 4C2). Most of the error remained within ±0.2 mN, and the statistical distribution of the error (Fig. 4C3) exhibited a near-Gaussian shape, indicating good stability and repeatability. In square-wave loading experiments (Fig. 4D1), the abrupt transitions in force posed a challenge for real-time response. The IPEM showed slight hysteresis during sharp transitions but still followed the overall trend of the commercial sensor. Error analysis (Fig. 4D2) revealed larger deviations at the edges but high consistency during the steady-state phases. The error distribution (Fig. 4D3) was more scattered compared to sinusoidal loading, primarily ranging between –1 and 2 mN, suggesting that the piezoelectric sensor retained the ability to detect sudden dynamic forces.

To further evaluate the accuracy of the system under various force amplitudes and frequencies, the piezoelectric and commercial force sensors were compared under periodic loading (Section S4.2 and Fig. S4 provide a detailed description). Table 1 summarizes key error metrics across different test conditions, including mean absolute error (MAE), standard deviation (SD), root-mean-square error (RMSE), and mean absolute percentage error (MAPE).

**Table 1.** Force comparison between piezoelectric sensor and commercial force sensor under different motion conditions

Frequency/Force	Force/Frequency	MAE/mN	SD/mN	RMSE/mN	MAPE
0.5 Hz	±3 mN	0.10	0.12	0.12	3.06%
	±6 mN	0.14	0.18	0.18	2.17%
	±30 mN	0.26	0.28	0.33	0.80%
	±60 mN	0.21	0.24	0.26	0.32%
±60 mN	0.6 Hz	0.26	0.31	0.31	0.43%
	0.7 Hz	0.26	0.30	0.30	0.42%
	0.8 Hz	0.42	0.44	0.47	1.30%
	1 Hz	0.16	0.20	0.20	0.26%

Under low-frequency loading conditions (0.5 Hz), as the force amplitude increased (from ±3 to ±60 mN), measurement errors slightly increased—MAE rose from 0.10 to 0.21 μm, and RMSE from 0.12 to 0.26 μm. However, MAPE decreased significantly from 3.06% to 0.32%, suggesting that relative error declined with increasing load. This reflects the fact that the piezoelectric sensor is more susceptible to noise under small signals but exhibits improved stability at moderate loads. Further analysis of frequency variation under a fixed force amplitude (±60 mN) from 0.6 to 1 Hz showed that in the 0.6 to 0.7 Hz range, MAE and RMSE remained stable between 0.26 and 0.31 μm, with MAPE below 0.43%, indicating reliable tracking performance in the low- to mid-frequency range. However, at 0.8 Hz, errors increased noticeably—RMSE reached 0.47 μm and MAPE rose to 1.30%, suggesting that faster loading compromised response accuracy, possibly due to delayed internal charge transfer within the piezoelectric material. Interestingly, at 1 Hz, the error decreased again (RMSE: 0.20 μm), possibly due to stabilization of the loading cycle. To verity real-time performance, the system exhibits a response time of approximately 8.5 ms (accounting for charge amplification, low-pass filtering, and ADC sampling) and a dynamic bandwidth of approximately 14.7 Hz, enabling effective capture of puncture transients without significant delay.

Overall, the piezoelectric sensor exhibited MAE below 0.3 μm and MAPE below 1% in most conditions, demonstrating excellent stability and accuracy. Its performance under moderate frequencies and loading amplitudes was comparable to that of commercial sensors, highlighting its potential for practical application in dynamic micro-force-sensing scenarios. The piezoelectric force-sensing component offers favorable linearity, high sensitivity, and low dynamic error, making it suitable for applications such as neural interface systems that demand high resolution and fast response.

### Puncture experiment on soft tissue models

#### Gelatin phantom test

**Fig. 5.**
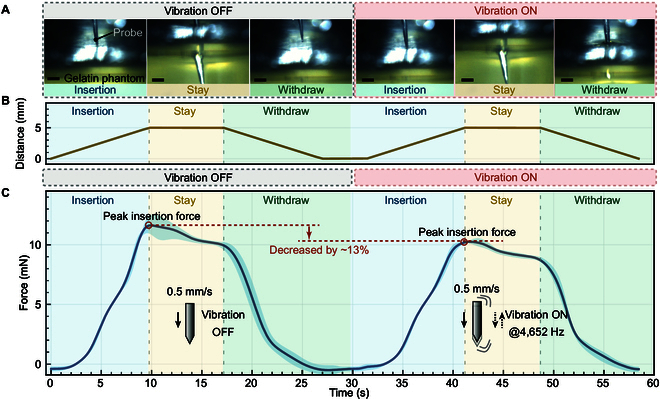
(A) Sequential photographs of the puncture process in gelatin phantom. (B) Insertion force with and without vibration. (C) The distance of the probe moves during the puncture process. All scale bars: 500 μm

To evaluate the effectiveness of the developed piezoelectric puncture system in simulated soft tissue environments, a series of controlled puncture experiments were conducted on gelatin phantoms (see Fig. S5 for the experimental setup). The gelatin samples were prepared with a concentration of 4.8% (w/v), corresponding to a Young’s modulus of approximately 0.5 to 2 kPa, mimicking the mechanical characteristics of soft biological membranes (Section S5.1 provides a detailed description). In each trial, the probe followed a fixed insertion trajectory: it was inserted into the gelatin at a constant speed of 0.5 mm/s to a depth of 5 mm, held stationary within the phantom for 5 s, and then retracted at the same speed. The experimental sequence is shown in Fig. 5A, and the probe movement process is shown in Fig. 5B. The experiment consisted of 2 phases: in the first phase, the vibration was turned off throughout the entire insertion–retraction cycle; in the second phase, the same insertion protocol was repeated under vibration-assisted mode. Between the 2 phases, the gelatin was manually shifted laterally to ensure the probe punctured an untested region. In both conditions, force signals were continuously recorded via the integrated IPEM throughout the entire motion cycle.

Under the nonvibration condition (*n* = 4), the puncture force was gradually increased, and after reaching the maximum value, a rapid drop in force occurred, characterizing the moment when the probe tip broke through the surface film of the gelatin phantom. At this time, the peak puncture force was $F_{\text{non}-\mathrm{vib}}=(11.95\pm 0.63)$ mN, showing an obvious “peak-drop” feature (Fig. 5C). After the subsequent activation of vibration, the force curve as a whole showed a relatively low puncture resistance, and the peak puncture force decreased significantly to $F_{\max}=(10.44\pm 0.22)$ mN, with a peak reduction amplitude of approximately 13%. In addition, the steepness of the force curve is also alleviated, indicating that the vibration makes the puncture process smoother. Statistical analysis using a paired *t* test confirmed a significant reduction in peak puncture force with vibration (*P* < 0.05), with a 95% confidence interval for the mean difference of (0.03, 3.00) mN. These results confirm that high-frequency axial vibration can assist in reducing the force required for penetrating viscoelastic media such as gelatin, validating the proposed mechanism of vibration-induced stress localization and fracture facilitation.

To assess thermal safety, a gelatin phantom experiment was conducted using infrared thermography. Initially, the IPEM and phantom temperatures were recorded (Fig. 3F), showing a maximum of 27.6 °C at the IPEM edge and 24.1 °C at the phantom center. After activating vibration for 10 min with 5-mm insertion in the tungsten probe, temperatures rose to 28.1 °C at the IPEM–probe junction and 24.2 °C at the phantom center (Fig. 3G), indicating a maximum increase of 0.5 °C in the IPEM and 0.1 °C in the phantom, well below the neural damage threshold of 43 °C [34].

#### In vivo mouse brain insertion

To further validate the functionality and biological applicability of the vibration-assisted puncture system, in vivo experiments were performed on anesthetized mice (*n* = 3), targeting the penetration of the soft meningeal layer covering the cortical surface. All experimental animal procedures are carried out in conformity with the laboratory animal protocol approved by the Institutional Animal Care and Use Committee (IACUC) at the Shanghai Jiao Tong University, Shanghai, China. The protocol number is A2024344-001.

A standard craniotomy procedure was carried out to expose the motor cortex. After the skull was carefully removed, the dura mater was excised under a surgical microscope, fully exposing the underlying pia mater, which remains as the primary soft tissue barrier. The pia mater, a thin and highly vascularized membrane, poses a greater challenge for low-damage penetration due to its elasticity and close adhesion to the cortical surface (Section S5.2 provides a detailed description).

**Fig. 6.**
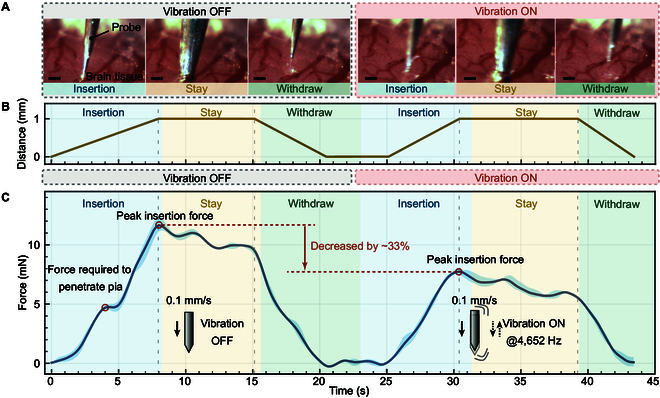
(A) Photographs of insertion stages in live tissue. (B) Insertion force with and without vibration. (C) The distance of the probe moves during the puncture process. All scale bars: 200 μm.

Beyond simple penetration, the experimental objective also included mimicking the implantation of a flexible neural electrode carried by a tungsten guide probe into the cortical tissue, followed by detachment of the electrode and withdrawal of the probe (the experimental sequence is shown in Fig. 6A). This process simulates a typical implantation procedure for minimally invasive brain–machine interfaces, wherein preserving electrode position and minimizing cortical trauma during retraction are critical.

The insertion protocol was identical to the gelatin phantom test. The probe was driven vertically at a constant velocity of 0.1 mm/s to a depth of 1 mm (including free travel before contact), held at maximum depth for more than 5 s, and then retracted (Fig. 6B). Two insertion conditions were compared: (a) without vibration and (b) with axial high-frequency vibration enabled. Between the 2 conditions, the probe was realigned to an adjacent unpierced cortical region to ensure independence of measurements. The piezoelectric sensing element recorded force responses continuously throughout the procedure (the experimental process and the pressure change curve can be found in Movie S1).

The force–time curves obtained from the in vivo trials show a clear distinction between the 2 conditions as shown in Fig. 6C. During nonvibrating insertion, a pronounced increase in force was observed before the moment of penetration, followed by a rapid drop, indicating rupture of the pia mater. In contrast, when vibration was applied, the force profile showed a reduced peak and smoother transition across the puncture phase. This reduction is attributed to dynamic stress concentration and micro-separation at the tissue interface induced by the high-frequency axial oscillation. Under nonvibration, the peak puncture force was (11.67 ± 0.44) mN, dropping to (7.80 ± 0.19) mN with vibration, representing a 33% reduction approximately. A paired *t* test showed that this difference was statistically significant (*P* < 0.05), with a 95% confidence interval for the mean difference of (2.97, 4.77) mN. In addition to the pia mater penetration force, the curves also reflect the insertion force within the cortical tissue after successful puncture, as well as the withdrawal resistance during probe retraction. Without vibration, cortical insertion was accompanied by gradually increasing resistive force, likely due to adhesion and tissue compression. In contrast, the vibrating mode reduced both the average insertion force and peak withdrawal resistance, supporting effective reduction of adhesion and friction at the probe–tissue interface. These observations are particularly relevant for electrode implantation scenarios, as lower insertion and withdrawal force implies less disturbance to the embedded electrode and surrounding cortex.

These findings reinforce the effectiveness of the proposed vibration-assisted puncture strategy under biologically relevant conditions. By lowering penetration and insertion forces and improving electrode detachment smoothness, the system holds promise for minimally invasive neural interfacing applications, especially where precision and tissue preservation are critical.

### Discussion

#### System advantages and limitations

Precise control over tissue penetration is critical in numerous biomedical applications, including intracortical neural probe insertion, retinal microsurgery, and cell membrane puncture. However, traditional insertion mechanisms either lack real-time force feedback or rely on bulky external sensors that compromise spatial resolution and integration. This work addresses those limitations by proposing a piezoelectric module that combines axial micro-vibration for puncture facilitation with direct force sensing via the piezoelectric effect, achieving a compact, self-sensing actuator. Compared to traditional ultrasonic probes or external load cells, this design offers several advantages: The actuator and sensor are combined using a back-to-back configuration of the same thin and lightweight piezoelectric element, eliminating the need for separate sensing modules and reducing system complexity. Force sensing occurs at the point of contact, avoiding signal loss or distortion associated with remote sensing. Unlike conventional ultrasonic or rotary mechanisms, the axial vibration approach reduces shear-induced tissue damage and maintains insertion path stability. In vivo experiments on anesthetized mice confirmed the ability of the system not only to pierce the pia mater but also to execute a realistic insertion–detachment sequence, demonstrating its potential as a tool for minimally traumatic electrode implantation in BCI applications. The force profiles recorded during both insertion and retraction phases revealed key transitions—e.g., pia rupture, cortical engagement, and detachment—that can be used to guide intelligent control strategies in future work. Our 33% in vivo force reduction aligns with vibration-assisted benchmarks, exceeding the 24.43% frictional reduction in ex vivo brain tissue reported by Wu et al. [33] but below a factor of 2 to 3 achieved via ultrasonic actuation in live mouse cortex by Jeong et al. [14], highlighting trade-offs in frequency and amplitude optimization. The in situ piezoelectric sensing achieves sub-millinewton resolution akin to the 0.314 mV/kPa tactile sensors in Zhou et al.’s [15] mosquito-inspired probe, but integrates actuation for vibration-assisted insertion, potentially enabling more adaptive control in heterogeneous tissues.

Despite these strengths, several limitations must be objectively acknowledged. The system’s sensitivity to electrical noise, as observed in dynamic tests, could amplify errors in noisy surgical environments without advanced filtering. Temperature and humidity variations may influence piezoelectric performance; for PZT materials, temperature variations affect piezo gain and frequency response (stable around room temperature but degrading at extremes), while high humidity may cause dielectric degradation and leakage currents, potentially impacting sensing accuracy in uncontrolled settings [35]. The current resonant frequency is optimized for soft tissues but may require tuning for stiffer media like cornea, where impedance mismatch could reduce efficiency. Additionally, while force reduction is significant, it was tested on limited samples, and variability in tissue heterogeneity could cause unpredicted spikes. The response time is adequate for low-speed insertion but may lag in ultrafast procedures.

#### Future directions in closed-loop control

While this study focuses on forward modeling and system validation, it lays the groundwork for subsequent integration of closed-loop feedback and adaptive control algorithms. In this study, the theoretical model assumed tissue homogeneity, but brain tissue heterogeneity (e.g., blood vessels and fiber bundles) can cause local force spikes during insertion, as reported in in vivo studies (e.g., the average insertion force required to penetrate the vessel is approximately 4 times higher than that of the basal brain tissue) [15]. Our system integrates sensors that can detect such transients in real time and may issue heterogeneity alerts without generating erroneous behavior. To mitigate these risks, preoperative planning can identify and avoid critical structures such as blood vessels. Our previous work introduces a fused photoacoustic and operating microscopic imaging system with cross-modality representation and registration network [36], enabling robot-controlled preoperative mapping of insertion sites to minimize encounters with heterogeneities, thereby reducing potential sensor fouling, collateral tissue damage, and insertion complications. Nevertheless, it is possible to incorporate force thresholds to pause or adjust parameters when spikes are detected, thereby reducing the risk of damage or dislocation. Therefore, future studies will also explore the following research directions, which include real-time modulation of vibration amplitude and frequency based on sensed force feedback; dynamic adjustment of insertion speed and trajectory to adapt to heterogeneous tissue properties; and extension of the system for multichannel or array-based microelectrode implantation. For instance, a proportional–integral–derivative controller could maintain tip forces below a predefined threshold by modulating vibration intensity during the pre-puncture phase, or model predictive control can be used to anticipate tissue rupture events using probe–tissue dynamics models to proactively optimize parameters and avoid overshoot. Furthermore, hybrid force/position control strategies could be used to combine axial position tracking from the robotic arm with inner-loop force compensation for synchronous regulation. These enhancements would expand the system’s applicability to complex scenarios like multilayer tissue penetration, with subsequent experimental validation in larger studies ensuring clinical translational potential.

#### Mechanical compatibility across tissue types

A critical aspect for broader adoption in diverse biomedical applications, such as tumor tissue biopsy, corneal puncture surgery, flexible neural electrode implantation, and single-cell puncture as shown in Fig. 1C, is analyzing mechanical compatibility, particularly impedance mismatch between the probe and varying tissue types. Different biological tissues exhibit distinct mechanical properties that influence penetration dynamics: brain tissues, relevant for neural electrode implantation, are soft and viscoelastic with Young’s moduli typically ranging from 1 to 100 kPa and nonlinear stress–strain responses, making them prone to deformation but susceptible to shear damage from mismatch-induced vibrations [37]. Tumor tissues vary widely, often stiffer (up to several kilopascals higher than surrounding healthy tissue) due to fibrosis, leading to heterogeneous impedance that could cause inefficient energy transfer and uneven rupture [38]. Corneal tissues are denser and more elastic (Young’s modulus: 0.1 to 10 MPa), with higher stiffness potentially resulting in increased resistance during puncture that amplifies insertion forces [39]. Single-cell membranes are extremely compliant (moduli in the pascal to kilopascal range), where small-scale mismatches might lead to excessive local stress and lysis [40]. Impedance mismatch—arising from these differences in stiffness, density, and viscoelasticity—can reduce vibration energy propagation efficiency, increasing peak forces or causing off-axis trauma. To address this issue specifically, future analyses could include tuning vibration frequency and amplitude via finite element simulations to match the target tissue’s resonant properties—for softer brain tissues, lower frequencies might minimize reflections and enhance controlled rupture. For tumors, adaptive algorithms could incorporate real-time force feedback to adjust parameters based on detected heterogeneity. For denser corneal layers, higher amplitudes could be used to overcome stiffness for improved rupture efficiency. For single-cell applications, ultralow-amplitude micro-vibrations paired with sharper probe tips would reduce mismatch at the small scale. Empirical characterization using our self-sensing module to measure force–displacement profiles across phantoms mimicking these tissues would further optimize the system, with preliminary simulations validating compatibility before in vivo trials to ensure minimal damage and maximal precision.

#### Integration challenges with surgical robotics

For integration with surgical robotic systems as emphasized in our robotic arm setup (Fig. 1A), key challenges include ensuring module sterilization compatibility, enabling rapid probe exchange, and suppressing electrical noise in vivo. Piezoelectric materials like PZT may not withstand high-temperature steam autoclaving due to potential degradation of piezo properties, necessitating alternatives such as low-temperature plasma or ethylene oxide sterilization for biocompatibility. Designs for rapid probe exchange could incorporate modular quick-connect mechanisms, such as magnetic or geared adapters, to facilitate sterile swaps during procedures without compromising precision. In vivo electrical noise from biological signals or electromagnetic interference can be mitigated through shielding, grounding, and low-pass filtering in the charge amplifier circuit. Addressing these in future iterations will enhance the system’s robustness for clinical microsurgery.

## Conclusion

This study introduces a novel piezoelectric penetration system that integrates vibration-assisted actuation with in situ force sensing, addressing key challenges in low-damage, high-precision implantation tasks for bio-interfacing applications. The system was designed to minimize insertion force through axial high-frequency vibration while simultaneously enabling direct measurement of puncture and insertion forces via the direct piezoelectric effect. Through a series of experiments including static calibration, dynamic force tracking, gelatin phantom tests, and in vivo mouse brain penetration, the system demonstrated the following characteristics: (a) high linearity and sensitivity in force sensing, achieving an *R*^2^ of 0.9998 in static calibration and real-time tracking accuracy comparable to commercial sensors; and (b) effective force reduction during vibration-assisted penetration, with clear reductions in peak puncture force, insertion force, and withdrawal resistance. In vivo experiments conducted on anesthetized mice confirmed that turning on vibration reduced the maximum insertion force by 33% compared to that without vibration. The system shows robust performance across various loading conditions, maintaining low error rates under both sinusoidal and abrupt force input profiles. Functional demonstration of electrode implantation shows that the probe can successfully mimic the process of pia mater penetration, cortical insertion, electrode release, and probe withdrawal with force sensing throughout.

## Data Availability

The data used to support the findings of this study are included in the article.
