# Supplementary material for: Integrated Piezoelectric Vibration and In Situ Force Sensing for Low-Trauma Tissue Penetration
Source: Cyborg Bionic Syst. 2025 Oct 21;6:0417. doi: 10.34133/cbsystems.0417 (PMC12538090; doi:10.34133/cbsystems.0417)
Supplement: Supplementary 1 — Supplementary Text Tables S1 and S2 Figs. S1 to S5 Movie S1 [file cbsystems.0417.f1.zip › Supplementary material.pdf]

Integrated Piezoelectric Vibration and In-Situ Force Sensing for Low-Trauma Tissue Penetration
Supplementary Materials

Bingze He[1,2], Yao Guo[1,2*], and Guang-Zhong Yang[1,2*]

[1]Institute of Medical Robotics, School of Biomedical Engineering, Shanghai Jiao Tong University, Shanghai, China.
[2]Shanghai Key Laboratory of Flexible Medical Robotics, Tongren Hospital, Institute of Medical Robotics, Shanghai Jiao Tong University, Shanghai, China.
[*]Address correspondence to: yao.guo@sjtu.edu.cn; gzyang@sjtu.edu.cn

1 Piezoelectric Constitutive Relations

The electromechanical coupling behavior of piezoelectric materials is governed by linear constitutive equations. For the PZT-5H material (poled along the 3-axis) used in this work, the three-dimensional constitutive relations are presented below.

1.1 Actuator Equation (Inverse Piezoelectric Effect)

The strain $\mathbf{S}$ generated by an applied electric field $\mathbf{E}$ is described by:

$$\mathbf{S} = \mathbf{s}^E\mathbf{T} + \mathbf{d}^t\mathbf{E} \tag{1}$$

In matrix form, this becomes:

$$\begin{bmatrix} S_1 \\ S_2 \\ S_3 \\ S_4 \\ S_5 \\ S_6 \end{bmatrix} = \begin{bmatrix} s_{11} & s_{12} & s_{13} & 0 & 0 & 0 \\ s_{12} & s_{22} & s_{23} & 0 & 0 & 0 \\ s_{13} & s_{23} & s_{33} & 0 & 0 & 0 \\ 0 & 0 & 0 & s_{44} & 0 & 0 \\ 0 & 0 & 0 & 0 & s_{55} & 0 \\ 0 & 0 & 0 & 0 & 0 & s_{66} \end{bmatrix} \begin{bmatrix} T_1 \\ T_2 \\ T_3 \\ T_4 \\ T_5 \\ T_6 \end{bmatrix} + \begin{bmatrix} 0 & 0 & d_{31} \\ 0 & 0 & d_{32} \\ 0 & 0 & d_{33} \\ 0 & d_{24} & 0 \\ d_{15} & 0 & 0 \\ 0 & 0 & 0 \end{bmatrix}^T \begin{bmatrix} E_1 \\ E_2 \\ E_3 \end{bmatrix} \tag{2}$$

where S_i ($i = \{1, \cdots, 6\}$) are the strain components (dimensionless), with S_1, S_2, and S_3 representing normal strains and S_4, S_5, and S_6 representing shear strains. T_j ($j = \{1, \cdots, 6\}$) are the stress components in Pa, s^E_{ij} is the elastic compliance matrix at constant electric field in $\mathrm{m^2\,N^{-1}}$, d_{kl} are

the piezoelectric strain coefficients in $\mathrm{m\,V^{-1}}$ or $\mathrm{C\,N^{-1}}$, and E_k $(k = \{1, \cdots, 3\})$ are the applied electric field components in $\mathrm{V\,m^{-1}}$.

1.2 Sensor Equation (Direct Piezoelectric Effect)

The electric displacement $\mathbf{D}$ generated by mechanical stress is given by:

$$\mathbf{D} = \mathbf{dT} + \boldsymbol{\varepsilon}^T\mathbf{E} \tag{3}$$

In matrix form:

$$\begin{bmatrix} D_1 \\ D_2 \\ D_3 \end{bmatrix} = \begin{bmatrix} 0 & 0 & 0 & 0 & d_{15} & 0 \\ 0 & 0 & 0 & d_{24} & 0 & 0 \\ d_{31} & d_{32} & d_{33} & 0 & 0 & 0 \end{bmatrix} \begin{bmatrix} T_1 \\ T_2 \\ T_3 \\ T_4 \\ T_5 \\ T_6 \end{bmatrix} + \begin{bmatrix} \varepsilon_{11} & 0 & 0 \\ 0 & \varepsilon_{22} & 0 \\ 0 & 0 & \varepsilon_{33} \end{bmatrix} \begin{bmatrix} E_1 \\ E_2 \\ E_3 \end{bmatrix} \tag{4}$$

where D_k $(k = \{1, \cdots, 3\})$ are the electric displacement components in $\mathrm{C\,m^{-2}}$, and ε_{ij}^T is the permittivity matrix at constant stress in $\mathrm{F\,m^{-1}}$.Table 1 summarizes the physical parameters of PZT-5H ceramics.

Table 1: Physical Parameters of the PZT-5H Ceramics

ε ($\times 10^{-9}$ F/m)	e (C/m^2)	C ($\times 10^{10}$ N/m^2)
$\begin{bmatrix} 7.65 & 0 & 0 \\ 0 & 7.65 & 0 \\ 0 & 0 & 6.40 \end{bmatrix}$	$\begin{bmatrix} 0 & 0 & -2.4 \\ 0 & 0 & -2.4 \\ 0 & 0 & 17.3 \\ 0 & 12.95 & 0 \\ 12.95 & 0 & 0 \\ 0 & 0 & 0 \end{bmatrix}$	$\begin{bmatrix} 14.3 & 7.85 & 7.85 & 0 & 0 & 0 \\ 7.85 & 14.3 & 7.85 & 0 & 0 & 0 \\ 7.85 & 7.85 & 11.5 & 0 & 0 & 0 \\ 0 & 0 & 0 & 2.6 & 0 & 0 \\ 0 & 0 & 0 & 0 & 2.45 & 0 \\ 0 & 0 & 0 & 0 & 0 & 2.45 \end{bmatrix}$

1.3 Charge Generation Mechanism

The total charge q in C generated by the piezoelectric stack under an axial force F_z is obtained by integrating the electric displacement D_3 over the electrode area A:

$$q = \iint_A D_3 \, dA = d_{33}F_z + d_{31} \iint (T_1 + T_2) \, dA \tag{5}$$

For the axisymmetric probe design, the transverse stresses T_1 and T_2 are related to the axial stress $T_3 = F_z/A$ through Poisson's ratio $\nu = 0.35$ for PZT-5H:

$$T_1 = T_2 = -\nu T_3 \tag{6}$$

The total charge can then be expressed as:

$$q = \left(d_{33} - 2\nu d_{31}\right) F_z = d_{\text{eff}} F_z \tag{7}$$

1.4 Temperature Effects and Compensation

The temperature dependence of PZT-5H's piezoelectric properties follows:

$$\frac{\Delta d_{33}}{d_{33}} = 0.004\,{}^{\circ}\mathrm{C}^{-1}, \quad \frac{\Delta \varepsilon_{33}^{T}}{\varepsilon_{33}^{T}} = 0.005\,{}^{\circ}\mathrm{C}^{-1} \quad (25 - 100\,{}^{\circ}\mathrm{C}) \tag{8}$$

2 Detailed Design Parameters of the IPEM

The IPEM is engineered to combine high-frequency axial vibration-assisted puncture with in-situ force sensing for low-damage insertion into soft biological tissues. The system comprises two piezoelectric ceramic discs (PZT), a tungsten probe, a copper substrate, and a mechanical clamping structure, all aligned along a central axis to ensure efficient force transmission and vibrational stability. The design prioritizes compactness, biocompatibility, and high sensitivity for biomedical applications such as neural electrode implantation and microsurgical procedures. Below, we detail the specifications of each component.

The piezoelectric elements are made of lead zirconate titanate (PZT), polarized along the thickness direction to operate in the d_{33} mode, maximizing axial displacement and force sensing sensitivity. Both the actuating and sensing PZT discs have a diameter of 10 mm and a thickness of 2 mm. The piezoelectric coefficient d_{33} is approximately $450\,\mathrm{pC\,N^{-1}}$, as measured for the specific batch used. Each PZT disc has a capacitance of 16 nF, ensuring sufficient charge generation for force sensing. The disc surfaces are coated with silver electrodes (10 µm thick), which are connected to external circuits via low-temperature soldering to ensure low contact resistance ($< 0.1\,\Omega$).

The tungsten probe, serving as the puncture tool, is fabricated from medical-grade tungsten with a Young's modulus of approximately 400 GPa, ensuring rigidity during insertion. The probe tip is machined into a conical shape with a half-cone angle $\theta_{hc} = 15°$, balancing puncture efficiency and minimal tissue damage. The probe shaft has a diameter of 500 µm and a length of 38 mm, with a tip radius of approximately 1 µm, as verified by scanning electron microscopy. The probe is rigidly bonded to the actuating PZT disc using high-strength epoxy resin (shear strength > 20 MPa), ensuring effective transmission of vibrational and contact forces.

The mechanical clamping structure consists of two stainless steel clamping rings (outer diameter 12 mm, inner diameter 8 mm, thickness 1 mm), which provide a preload force of approximately 50 N to eliminate micro-gaps between PZT discs and enhance vibration transmission. The rings are secured using four M2 bolts with a controlled torque of 0.5 N m to avoid overstressing the PZT ceramics. The inner surfaces of the rings are polished (surface roughness $Ra < 0.2$ µm) to minimize frictional interference with vibrational modes. The rings also serve as electrical contact surfaces, connected to the PZT electrodes via conductive gaskets.

A copper substrate, with a thickness of 0.13 mm, serves as the base for the assembly, providing mechanical stability and electrical grounding. The copper substrate is chosen for its high thermal and electrical conductivity, ensuring efficient heat dissipation during high-frequency operation and reliable electrical connections. Table 2 summarizes the properties of all the materials.

The IPEM assembly has a total mass of approximately 5 g and a compact height of 38 mm (including the probe), making it suitable for integration with a 6-DOF robotic arm or a micromanipulation platform. Biocompatibility is ensured by treating all tissue-contacting surfaces (probe and clamping ring exteriors) with medical-grade electrochemical polishing to reduce the risk of immune responses. The rigid probe and high-stiffness clamping design validate the assumption of a rigid probe in the mechanical models described in the main text, ensuring accurate force transmission and minimal compliance losses.

Table 2: Material Properties of Components

Material	Parts	Density (kg/m^3)	Young's modulus (GPa)	Poisson ratio
304	Clamp ring	7800	206	0.30
Tungsten	Probe	19300	400	0.28
Copper	Substrate	8960	110	0.34
PZT-5H	Actuating/Sensing disc	7500	60	0.31
Silver	Ceramic electrode	10490	83	0.37

3 Electronic circuit Design

The charge amplifier circuit is the cornerstone of the in-situ force sensing capability of the IPEM, designed to convert the minute charge signals generated by the piezoelectric sensing disc into measurable voltage signals with high fidelity and low noise. This section details the design, operation, and performance of the charge amplifier, followed by descriptions of the filtering, analog-to-digital conversion (ADC), digital-to-analog conversion (DAC), and power amplification stages.

3.1 Charge Amplifier

The charge amplifier employs the OPA128 ultra-low input bias current operational amplifier (Texas Instruments), renowned for its suitability in piezoelectric charge amplification due to its input bias current of less than 75 fA and high input impedance (10 TΩ). The design follows the official recommended circuit configuration for piezoelectric charge amplification, as outlined in the OPA128 datasheet, which uses a feedback network to integrate the input charge into a proportional output voltage. In this study, the charge amplification circuit is shown in Figure 1.

The piezoelectric sensing disc generates charge proportional to the applied mechanical stress, modeled as a charge source in parallel with a capacitance of approximately 16 nF (as specified in the PZT disc design). The charge amplifier circuit integrates this charge using a feedback capacitor $C_f = 10\,\text{nF}$ and a feedback resistor $R_f = 100\,\text{G}\Omega$. The transfer function of the circuit is given by:

$$V_{\text{out}}(t) = \frac{Q(t)}{C_f},$$

where $Q(t)$ is the input charge and V_{out} is the output voltage. The feedback resistor R_f sets the low-frequency cutoff to mitigate drift, calculated as:

$$f_c = \frac{1}{2\pi R_f C_f} \approx 0.0016\,\text{Hz},$$

3.2 Filtering Stage

The filtering stage utilizes the TLC14 operational amplifier configured as a second-order low-pass filter to remove high-frequency noise and crosstalk from the 4.5 kHz vibration drive signal. The filter is designed with a cutoff frequency of 15 Hz, selected to preserve the relevant force signal bandwidth

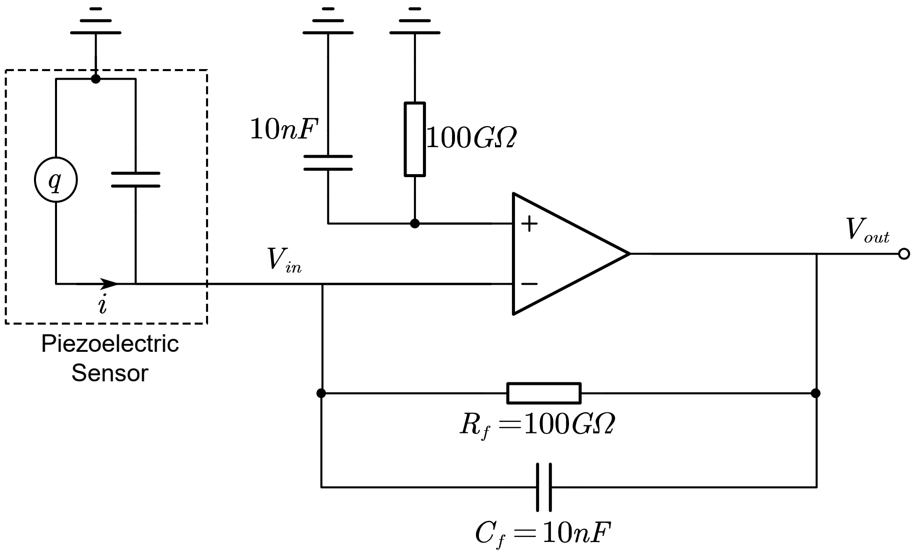

Figure 1: Circuit diagram of the charge amplifier using OPA128.

while attenuating higher frequencies. The filter employs a Sallen-Key topology with a damping ratio of 0.707 for a maximally flat response, achieved through appropriate resistor and capacitor values. The output of this stage feeds into the ADC for digitization.

3.3 Analog-to-Digital Conversion (ADC)

The analog-to-digital conversion is performed using the AD7606, a 16-bit simultaneous sampling ADC from Analog Devices, capable of handling up to six analog input channels. This device operates at a maximum sampling rate of $200\,\mathrm{ks}^{-1}$, with a signal-to-noise ratio of 91 dB, ensuring high-resolution capture of the filtered force signal. The AD7606 is configured in its default mode with a ± 5 V input range, synchronized with the system clock to align with the $10\,\mathrm{ks}^{-1}$ sampling rate used in the experiments. Digital data is transmitted via a parallel interface to the host computer for real-time analysis, with a latency of less than 5 µs.

3.4 Digital-to-Analog Conversion (DAC)

The digital-to-analog conversion stage employs the AD9959, a high-speed DDS (Direct Digital Synthesis) DAC from Analog Devices, designed to generate the 4.5 kHz sinusoidal drive signal for the piezoelectric actuator. The AD9959 offers a 14-bit resolution and a maximum output frequency of 400 MHz, with a spurious-free dynamic range of 80 dB. It is programmed via a serial peripheral interface (SPI) to produce a stable 4.5 kHz signal with a programmable amplitude, which is then amplified to 80 V. The DAC output is low-pass filtered internally to reduce harmonic distortion before amplification.

3.5 Power Amplification

The power amplification stage boosts the DAC output to the required 80 V to drive the piezoelectric actuator at the desired 4.5 kHz frequency. The circuit(see Figure 2), depicted in the provided schematic, utilizes a complementary pair of NPN and PNP transistors (MJE15030 and MJE15031) configured in a Class AB push-pull amplifier topology to achieve high efficiency and low distortion. The input signal is buffered by an OPA454AIDDAG4 operational amplifier, which provides a high output current capability (150 mA). Feedback resistors (10 kΩ, 200 kΩ) and a capacitor (1 pF) stabilize the amplifier. The circuit operates with a ±50 V supply (VCC and VEE), delivering the necessary voltage swing with a bandwidth exceeding 100 kHz.

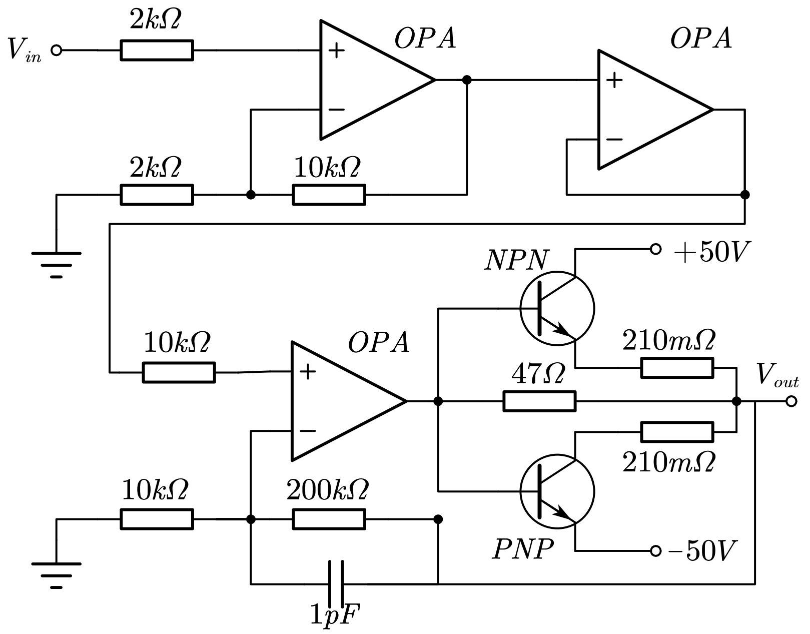

Figure 2: Circuit diagram of the power amplification stage.

The power amplifier is designed with thermal management in mind, incorporating a heat sink on the transistor pair to handle the 10 W dissipation at full load. The output is coupled to the piezoelectric actuator via a high-voltage capacitor to block DC components, ensuring safe operation.

4 Data Analysis Methods

4.1 Piezoelectric Sensor Calibration Experiment

The calibration of the piezoelectric sensor is conducted to establish the relationship between the mechanical force applied to the IPEM and the resulting voltage output, ensuring accurate force sensing. The experimental setup mirrors the "Performance Evaluation of Piezoelectric Force-Sensing Component" described in the main text, utilizing the 6-DOF robotic arm for precise displacement control witch is a Stewart platform parallel 6-DOF robotic arm, featuring a Z-axis repeatability of 1 µm and a Z-axis workspace of 40 mm.

The calibration process involves mounting the IPEM on the robotic arm, with a calibrated spring mechanism fixed to the probe tip to convert applied displacement into a measurable force. The robotic arm applies incremental displacements along the Z-axis, with each step of 1 mm, totaling seven steps from 0 mm to 7 mm. After each step, the system pauses for approximately 10 s to allow the spring and IPEM to stabilize, minimizing dynamic effects. A commercial force sensor is positioned in series with the IPEM to provide reference force measurements, while the piezoelectric sensor's output voltage is recorded after signal conditioning through the charge amplifier circuit.The curves recorded by the two sensors are shown in Figure 3.

Data collection occurs at a sampling rate of 10 ks^{-1} over the 10 s stabilization period for each step. The voltage output from the piezoelectric sensor and the corresponding force values from the commercial sensor are extracted for each of the eight displacement steps. For each step, the platform period (the stable portion of the 10 s interval, typically the last 5 s) is identified, and the mean values of both the force and voltage are calculated across all data points within this period. This results in 7 pairs of mean force and mean voltage values.

The calibration curve is obtained by performing a linear least-squares fit to these eight mean value pairs, using the model $V = k \cdot F + b$, where V is the voltage output (V), F is the force (mN), k is the sensitivity (mV/mN), and b is the offset (mV). The goodness of fit is evaluated using the coefficient of determination (R^2), and the uncertainty in k and b is estimated from the residual standard error. This process yields a sensitivity of 9.3 mV/mN and an R^2 of 0.9998, as reported in the main text, validating the sensor's linearity and precision.

4.2 Dynamic Force Sensing Tests

Dynamic force sensing tests are conducted to evaluate the IPEM's response to varying force profiles, comparing its performance against a commercial force sensor. The tests involve applying sinusoidal waveforms with frequencies ranging from 0.5 Hz to 1 Hz and amplitudes from ±3 mN to ±60 mN. These dynamic loads are generated using the robotic arm, with the IPEM and a reference commercial force sensor recording the force responses simultaneously(see as Figure 4). The error is defined as the pressure value of the commercial sensor at the same moment minus the pressure value of the piezoelectric sensor.

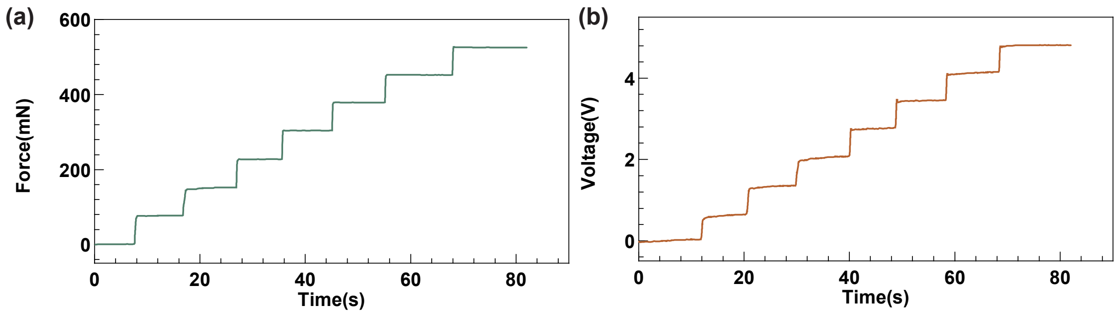

Figure 3: Calibration curves of pressure sensors and piezoelectric sensors.(a) The pressure curve measured by the pressure sensor. (2) The voltage curve of the piezoelectric sensor signal after conditioning.

5 Experimental Setup

The experimental setup is designed to validate the performance of the IPEM in simulated and biological environments. This section details the preparation and execution of gelatin phantom tests, in vivo mouse brain experiments.

5.1 Gelatin Phantom Test

The gelatin phantom test simulates the mechanical properties of soft biological tissues to evaluate the IPEM's vibration-assisted puncture and force sensing capabilities. Gelatin phantoms are prepared using food-grade gelatin (Sigma-Aldrich) at a concentration of 4.8% (w/v). The preparation involves dissolving gelatin powder in deionized water at 60 °C, stirring until fully mixed, and pouring the solution into a cylindrical mold with a diameter of 50 mm and a thickness of 20 mm. The mold is then cooled at 4 °C for 12 h to solidify. The resulting phantom exhibits a Young's modulus of 0.5 kPa to 2 kPa. **This range approximates established properties of mouse meningeal tissues, such as the pia mater and surrounding cortex, with Young's moduli reported as 0.03-12 kPa[1].** To prevent drying, the phantom surface is covered with a thin plastic film, and it is allowed to equilibrate at room temperature (25 °C) for 30 min prior to testing.

The IPEM is mounted on the robotic arm, integrated with a microscope (10x objective, resolution 1 μm) for real-time observation. The experimental setup is shown in Figure 5. The probe is inserted into the gelatin phantom at a constant speed of 0.5 mm s^{-1} to a depth of 5 mm, held stationary for 5 s, and then retracted at the same speed. Between trials, the phantom is manually shifted laterally by 5 mm to ensure the probe punctures an untested region. Force signals are continuously recorded via the IPEM's charge amplifier circuit at a sampling rate of 10 ks^{-1}.

5.2 In Vivo Mouse Brain Experiment

The in vivo experiment assesses the IPEM's performance in a biological context, specifically targeting the penetration of the mouse brain's pia mater. The study is conducted on adult BalbC mice (weight

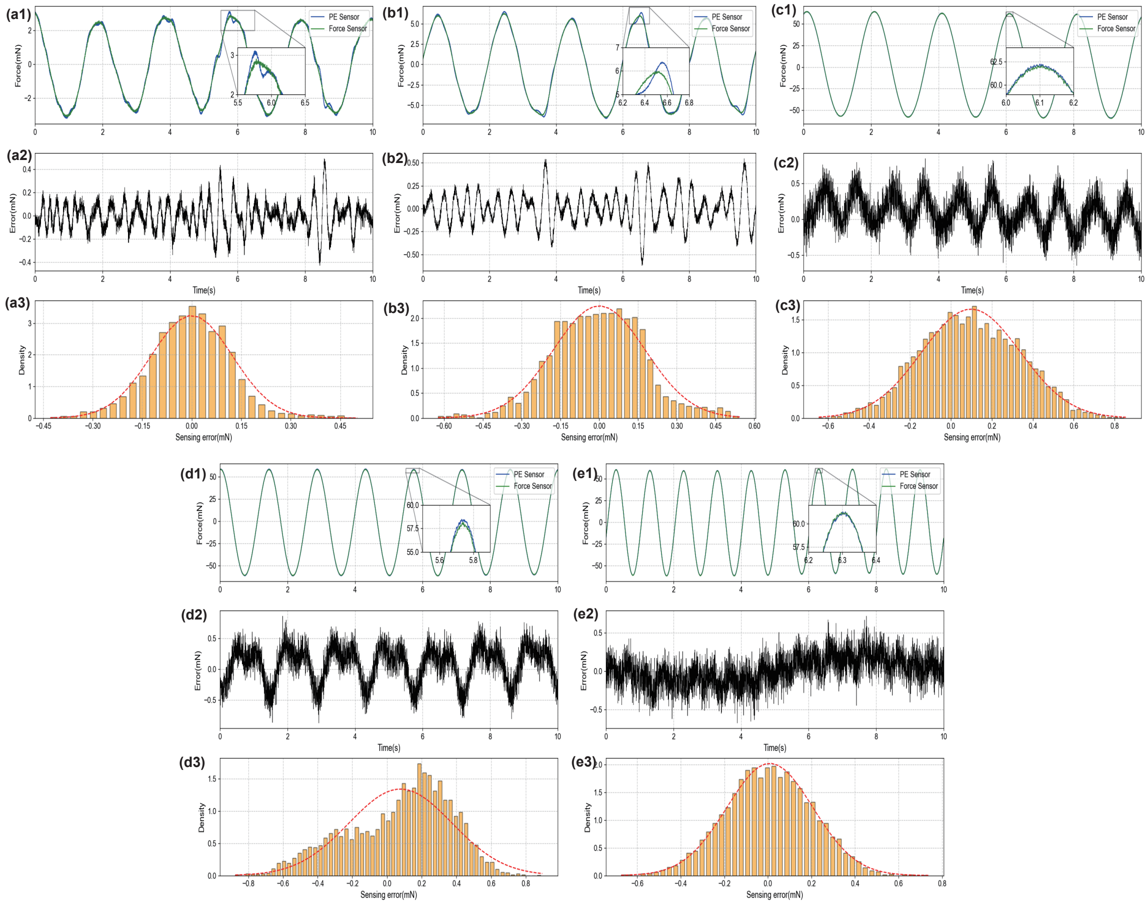

Figure 4: Force curves, errors, and error distributions of commercial force sensors and IPEM under sinusoidal force measurements.(a1)-(a3):Under 0.5Hz,6mN condition; (b1)-(b3):Under 0.5Hz,12mN condition; (c1)-(c3):Under 0.5Hz,120mN condition; (d1)-(d3):Under 0.7Hz,120mN condition; (e1)-(e3):Under 1Hz,120mN condition.

20 g to 25 g, male and female) following institutional animal ethics committee approval (The protocol number is No. A2024344-001). Anesthesia is maintained with inhaled isoflurane (1.5–2%), and body temperature is regulated at 37 °C using a heating pad.

A standard craniotomy is performed to expose the motor cortex, involving the removal of a 3 mm × 3 mm section of the skull under a surgical microscope. The dura mater is carefully excised, leaving the pia mater as the primary soft tissue barrier. The IPEM, mounted on the same robotic arm, executes an insertion protocol identical to the gelatin phantom test: the probe is driven vertically at 0.1 mm s^{-1} to a depth of 1 mm (including free travel before contact), held for 5 s, and then retracted. Between conditions (with and without vibration), the probe is realigned to an adjacent unpierced cortical region (spaced 1 mm apart). Force responses are recorded continuously using the piezoelectric sensing element, synchronized with microscopic imaging to document the puncture process.

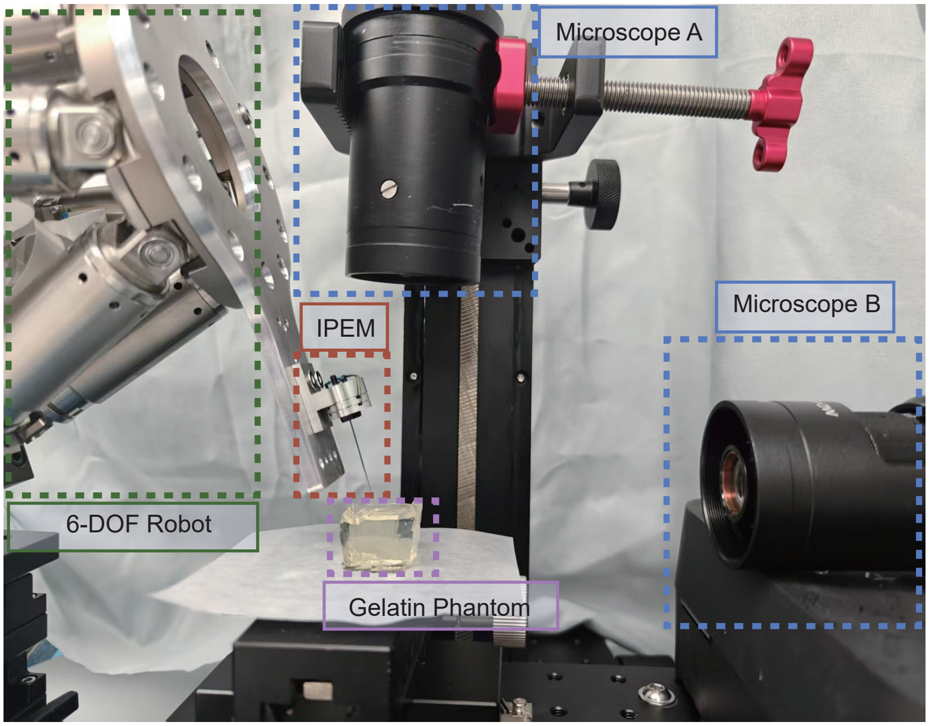

Figure 5: The experimental setup for the puncture test using gelatin phantom.

References

1. Exton J, Higgins JM, and Chen J. Acute brain slice elastic modulus decreases over time. Scientific Reports 2023;13:12826.
